# High‐Entropy Metal–Organic Frameworks for Highly Reversible Sodium Storage

**DOI:** 10.1002/adma.202101342

**Published:** 2021-07-09

**Authors:** Yanjiao Ma, Yuan Ma, Sören Lukas Dreyer, Qingsong Wang, Kai Wang, Damian Goonetilleke, Ahmad Omar, Daria Mikhailova, Horst Hahn, Ben Breitung, Torsten Brezesinski

**Affiliations:** ^1^ Institute of Nanotechnology Karlsruhe Institute of Technology (KIT) Hermann‐von‐Helmholtz Platz 1 76344 Eggenstein‐Leopoldshafen Germany; ^2^ Leibniz Institute for Solid State and Materials Research (IFW) Dresden Helmholtzstr. 20 01069 Dresden Germany; ^3^ Joint Research Laboratory Nanomaterials – Technische Universität Darmstadt and Karlsruhe Institute of Technology (KIT) Otto‐Berndt‐Str. 3 64206 Darmstadt Germany; ^4^ Helmholtz Institute Ulm (HIU) for Electrochemical Energy Storage Helmholtzstr. 11 89081 Ulm Germany

**Keywords:** gassing behavior, high‐entropy materials, Prussian blue analogues, secondary batteries, sodium‐ion cathodes

## Abstract

Prussian blue analogues (PBAs) are reported to be efficient sodium storage materials because of the unique advantages of their metal–organic framework structure. However, the issues of low specific capacity and poor reversibility, caused by phase transitions during charge/discharge cycling, have thus far limited the applicability of these materials. Herein, a new approach is presented to substantially improve the electrochemical properties of PBAs by introducing high entropy into the crystal structure. To achieve this, five different metal species are introduced, sharing the same nitrogen‐coordinated site, thereby increasing the configurational entropy of the system beyond 1.5R. By careful selection of the elements, high‐entropy PBA (HE‐PBA) presents a quasi‐zero‐strain reaction mechanism, resulting in increased cycling stability and rate capability. The key to such improvement lies in the high entropy and associated effects as well as the presence of several active redox centers. The gassing behavior of PBAs is also reported. Evolution of dimeric cyanogen due to oxidation of the cyanide ligands is detected, which can be attributed to the structural degradation of HE‐PBA during battery operation. By optimizing the electrochemical window, a Coulombic efficiency of nearly 100% is retained after cycling for more than 3000 cycles.

## Graphical abstract

A high‐entropy Prussian blue analogue, Na*_x_* (FeMnNiCuCo)[Fe(CN)_6_], is developed as a robust host structure for reversible sodium insertion. Battery cells using the new cathode active material show significantly improved cycling performance and stability, among others due to its nearly zero‐strain operation.

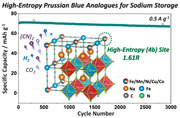

## 1 Introduction

In recent times, the introduction of high entropy into various materials for different applications has sparked increasing interest among researchers and promoted the rapid development of a series of single‐phase multicomponent (equimolar) materials.^[^ ^1^, ^2^, ^3^, ^4^ ^]^ In disordered multicomponent systems, large configurational entropy is commonly considered to stabilize the crystal structure, transmitting the high‐entropy (HE) effects, namely, entropy‐driven stabilization and the associated “cocktail” effects arising from cation mixing, and fostering their chemical and structural diversity.^[^ ^1^, ^4^, ^5^ ^]^ Within the past few years, a large number of high‐entropy materials (HEMs), represented first by high‐entropy alloys (HEAs)^[^ ^1^, ^5^, ^6^, ^7^, ^8^ ^]^ and later by high‐entropy oxides (HEOs),^[^ ^3^, ^9^, ^10^, ^11^, ^12^, ^13^ ^]^ have been utilized in a broad range of applications, including environmental protection, electrochemical energy storage, and thermoelectric and catalytic applications. Among battery materials, several recent reports have shown that the introduction of high entropy can substantially improve cycling performance, for instance, in HEOs and high‐entropy oxyfluorides (HEOFs).^[^ ^9^, ^10^, ^14^, ^15^, ^16^, ^17^, ^18^, ^19^, ^20^, ^21^, ^22^, ^23^, ^24^ ^]^ In a previous study by our group, rock‐salt (Co_0.2_Cu_0.2_Mg_0.2_Ni_0.2_Zn_0.2_)O was proposed as a promising anode material for lithium‐ion batteries (LIBs), with a unique entropy‐stabilized Li‐storage mechanism, guaranteeing the reversible conversion reaction and leading to improved cycling stability and Coulombic efficiency.^[^ ^9^ ^]^ Also, Hu and co‐workers reported on layered O3‐type HEO as an intercalation‐type cathode for sodium‐ion batteries (SIBs),^[^ ^10^ ^]^ which exhibited good long‐term cyclability and rate performance owing to entropy stabilization of the host matrix.

However, drawbacks of high‐entropy materials are that their preparation typically involves procedures with high energy costs, such as (high‐energy) ball milling or high‐temperature processing (>900 °C), and that they can be prone to phase separation (shown, e.g., for multimetallic nanoparticles).^[^ ^25^, ^26^ ^]^ Another restriction, specific to electrochemical applications, is that HEOs are reported to undergo unfavorable phase transitions during electrochemical cycling, which can make it difficult to resolve the individual effect of high entropy, as potential structural rearrangements cannot be fully ruled out.

A class of materials which has yet to attract attention in the field of high‐entropy battery materials are Prussian blue analogues (PBAs). PBAs belong to the family of metal–organic frameworks (MOFs). These materials are typically transition‐metal hexacyanoferrates with an open channel structure and abundant redox‐active sites. They have also been proposed as insertion cathodes for next‐generation batteries, especially SIBs.^[^ ^27^, ^28^, ^29^, ^30^, ^31^ ^]^ PBAs can be described using the chemical formula A_*x*_M[Fe(CN)_6_]_*y*_□_*m*_ · *n*H_2_O (with 0 < *x* ≤ 2, 0 < *y* ≤ 1, *y* + *m* = 1), or simplified as AM‐PBA, where A, M, and □ represent the mobile ions, transition‐metal ions, and vacancies, respectively. PBAs contain two redox‐active centers, the nitrogen‐coordinated high‐spin M^2 +/3 +^ and carbon‐coordinated low‐spin Fe^2 +/3 +^ sites,^[^ ^32^, ^33^, ^34^, ^35^ ^]^ enabling a theoretical specific capacity of 171 mAh g^−1^ for the sodium‐containing electrode material Na_2_Fe[Fe(CN)_6_]. Recently, it has been reported that the M position in this system can be substituted by other redox‐active transition metals, such as Mn, Ni, Cu, Co, V, and Cr, thus allowing modification of the electrochemical behavior.^[^ ^27^, ^28^, ^29^, ^30^, ^31^ ^]^

In this study, the high‐entropy approach is applied to the Prussian blue (PB) structure, utilizing metal cations previously reported in conventional single‐ or dual‐metal PBAs. The HE‐PBA material Na*_x_* (FeMnNiCuCo)[Fe(CN)_6_], where the different transition metals have been introduced to the M site in equimolarity, is presented. HE‐PBA shows a significant increase in reversible Na‐storage capability compared to medium‐entropy PBAs (ME‐PBAs) and conventional PBA with only Fe at the M site (Fe‐PBA), with all samples prepared through a simple coprecipitation method at room temperature.

## 2 Results and Discussion

**Figure 1:**
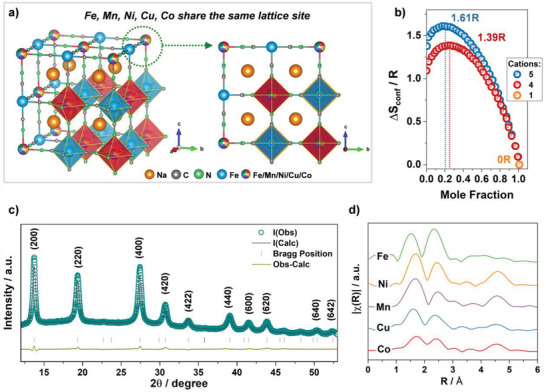
a) Schematic illustration of the crystal structure of HE‐PBA. b) Dependence of configurational entropy on the number of elements. c) XRD pattern of HE‐PBA and Rietveld plot for the refinement. d) Fourier transform of the EXAFS signals collected from the HE‐PBA material, for each metal edge, plotted with a *y*‐axis offset for better clarity.

The structure of HE‐PBA is illustrated in **Figure** **1**a, showing five equimolar cations, Fe, Mn, Ni, Cu, and Co, sharing the nitrogen‐coordinated M position (0.2 mol fraction each). The configurational entropy (Δ*S*_conf_) of this system is calculated to be 1.61R (Figure 1b), determined using the statistical thermodynamics derived definition for Δ*S*_conf_, see Equations (S1) and (S2) in the Supporting Information. A series of ME‐PBAs was also prepared, with one of the metal cations at the M position removed compared to HE‐PBA, resulting in a reduction of Δ*S*_conf_ to 1.39R (Figure 1b). The ME‐PBAs contain four metal cations at the M position (0.25 mol fraction each) and are denoted as ME‐PBA(‐Fe), ME‐PBA(‐Mn), ME‐PBA(‐Ni), ME‐PBA(‐Cu), and ME‐PBA(‐Co), representing the exclusion of Fe, Mn, Ni, Cu, and Co, respectively.

All prepared samples revealed a single‐phase cubic structure with the *Fm*−3*m* space group (Figure S1a, Supporting Information), with no impurities observed. A vacancy‐containing Na*_x_* Fe[Fe(CN)_6_]_1−_ *_y_* model (Inorganic Crystal Structure Database, ICSD coll. code 193354) was refined against the powder X‐ray diffraction (XRD) data, and the corresponding Rietveld refinement profile is shown in Figure 1c. The structural model used assumes the carbon‐coordinated Fe_1_ atoms occupy the 4a site, while the nitrogen‐coordinated Fe_2_, Mn, Co, Ni, and Cu atoms share the 4b site in equal molarity. The lattice parameters of HE‐PBA were determined to *a* = *b* = *c* = 10.271(1) Å, α = β = γ = 90° and *V* = 1083.45(40) Å^3^. This model suggests that the structure consists of linear chains of Fe_1_—C≡N—Mn/Ni/Cu/Co/Fe_2_—N≡C—Fe_1_ along the cube edges, as depicted in Figure 1a. More information about Rietveld refinement and the structural parameters are shown in Table S1 in the Supporting Information with detailed discussion.

X‐ray absorption spectroscopy (XAS) was performed to evaluate the local environment of each metal cation. The corresponding Fourier transforms of the extended X‐ray absorption fine structure (EXAFS) signals are shown in Figure 1d. The radial distribution is very similar, suggesting a homogeneous distribution of the cations at the mixed‐metal site. At each of the measured edges, three dominant features can be identified, which correspond to the M—N/C, M—C/N, and M—Fe shells. The Fourier transform for the Fe‐K edge is compounded by the Fe_2_ site (4b), leading to slight variation in comparison to the other edges. It should also be noted that small variations in the local environment are expected due to the different valences and ionic radii of the metal cations.

The structure was also examined by attenuated total reflection‐infrared spectroscopy (Figure S1b, Supporting Information). The observation of a strong band at 2081 cm^−1^ can be assigned to the stretching vibration of the C≡N group coordinated by Fe^2+^ and M^2+^, i.e., Fe^2+^—C≡N—M^2+^.^[^ ^8^, ^35^, ^36^, ^37^ ^]^ The vibrational bands at around 1607 and 3530 cm^−1^ correspond to the O—H bending modes of H_2_O, and the content of crystal water was determined to 7–8 wt% by thermogravimetric analysis (TGA, Figure S1c, Supporting Information). The weight loss at <120 °C was assigned to the evaporation of adsorbed H_2_O on the surface, while that in the temperature range of 120–200 °C can be attributed to the elimination of zeolitic water in the structure. The chemical formula of HE‐PBA was estimated as Na_1.19_(Fe_0.2_Mn_0.2_Ni_0.2_Cu_0.2_Co_0.2_)[Fe(CN)_6_]_0.79_□_0.21_·1.16H_2_O according to the results from TGA and inductively coupled plasma‐optical emission spectroscopy (ICP‐OES), see Table S2 in the Supporting Information.

**Figure 2:**
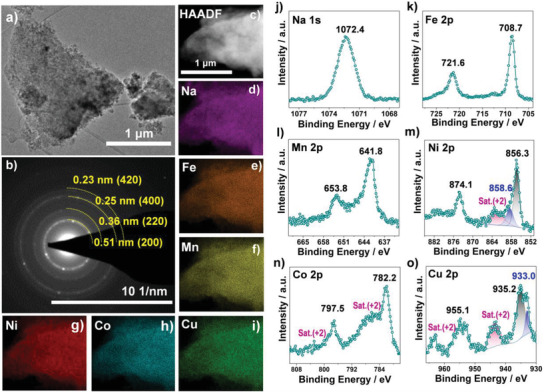
a) TEM image, b) SAED pattern, c) HAADF STEM image, and d–i) the corresponding EDS maps for Na (in purple), Fe (in orange), Mn (in yellow), Ni (in red), Co (in cyan), and Cu (in green) for HE‐PBA. j) Na 1s, k) Fe 2p, l) Mn 2p, m) Ni 2p, n) Co 2p, and o) Cu 2p XP core‐level spectra for HE‐PBA.

The structure of the HE‐PBA was further confirmed by transmission electron microscopy (TEM, **Figure** **2**a) and selected‐area electron diffraction (SAED, Figure 2b). Reflections corresponding to the (200), (220), (400), and (420) lattice planes of the PB structure were clearly observed. Scanning electron microscopy images of all samples are presented in Figures S2–S7 in the Supporting Information, accompanied by energy‐dispersive X‐ray spectroscopy (EDS) mapping for each metal component. Both HE‐PBA and the different ME‐PBAs showed bulk morphology with uniform distribution of the corresponding elements at the given resolution. EDS analysis further revealed that the M position cations are present in equimolar amounts, which is in good agreement with the ICP‐OES results (Table S2, Supporting Information). Note that the larger atomic weight fraction of Fe is because of the presence of carbon‐coordinated Fe_1_ in the PBA materials. The uniform distribution of Na, Fe, Mn, Ni, Cu, and Co in HE‐PBA was also corroborated by conducting high‐angle annular dark‐field scanning TEM (HAADF STEM) together with EDS characterization for spatially resolved element analysis (Figure 2c–i).

X‐ray photoelectron spectroscopy (XPS, see Figure S8, Supporting Information, for the survey spectrum) confirmed the presence of Na, Fe, Mn, Ni, Cu, and Co for HE‐PBA. A detailed analysis of each metal species is provided in Figure 2j–o. Note that the XPS results refer to the surface state of the metal species. The Na 1s spectrum showed a single peak at a binding energy of 1072.4 eV.^[^ ^38^ ^]^ Characteristic Fe 2p peaks appeared at 708.7 eV (Fe 2p_3/2_) and 721.6 eV (Fe 2p_1/2_), with a spin‐energy separation of 12.9 eV, thereby indicating 2+ oxidation state.^[^ ^37^, ^39^, ^40^, ^41^ ^]^ The peaks at 641.8 and 653.8 eV in the Mn 2p spectrum can be assigned to Mn 2p_3/2_ and Mn 2p_1/2_, respectively, in the divalent state.^[^ ^40^, ^41^, ^42^ ^]^ The Ni 2p spectrum showed major peaks at 856.3 eV (Ni 2p_3/2_) and 874.1 eV (Ni 2p_1/2_) together with satellite peaks at higher binding energies, which is typical of Ni^2+^.^[^ ^40^, ^42^ ^]^ Note that the minor peaks at 858.6 and 876.2 eV can be assigned to Ni^3+^.^[^ ^43^, ^44^ ^]^ Similarly, in the case of Co 2p and Cu 2p, (shake‐up) satellite peaks were detected at 786 and 802 eV and at 944 and 964 eV, respectively, indicating the presence of Co^2+[^ ^45^, ^46^ ^]^ and Cu^2+^.^[^ ^14^, ^47^ ^]^ Two additional peaks at 933.0 and 952.9 eV appeared in the Cu 2p spectrum, corresponding to Cu^1+^ 2p_3/2_ and Cu^1+^ 2p_1/2_, respectively.^[^ ^14^, ^47^ ^]^ This finding suggests that an internal redox reaction from Cu^2+^ to Cu^1+^ occurred during the synthesis, accompanied by partial oxidation of Ni^2+^ to Ni^3+^. Nevertheless, the average oxidation state of both the carbon‐coordinated Fe_1_ and nitrogen‐coordinated M (Fe_2_, Mn, Ni, Cu and Co) cations is 2+, as expected for the PB structure with linear chains of Fe^2+^—C≡N—M^2+^—N≡C—Fe^2+^.

**Figure 3:**
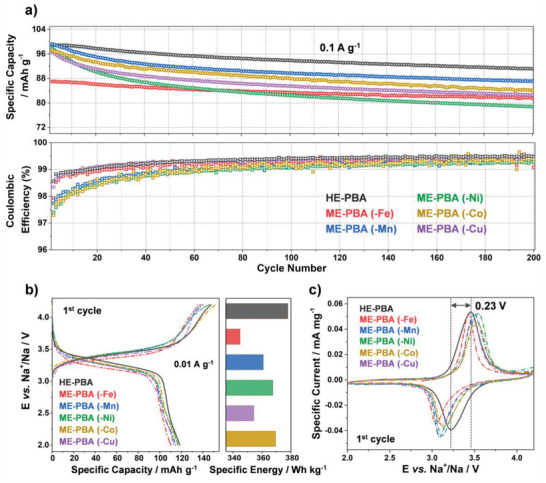
a) Galvanostatic cycling at 0.1 A g^−1^ of electrodes based on HE‐PBA (in black), ME‐PBA(‐Fe) (in red), ME‐PBA(‐Mn) (in blue), ME‐PBA(‐Ni) (in green), ME‐PBA(‐Co) (in dark yellow), or ME‐PBA(‐Cu) (in purple). Specific discharge capacities (top) and Coulombic efficiencies versus the cycle number (bottom) are shown (1st cycle is omitted for clarity). b) First‐cycle voltage profiles at 0.01 A g^−1^ (left) and comparison of specific energies (right). c) Initial cyclic voltammogram at 0.05 mV s^−1^ for HE‐PBA and the different ME‐PBAs.

After structural and chemical characterization confirming the successful introduction of the high‐entropy approach, we focused on the investigation of the impact of entropy on the electrochemical behavior. As mentioned above, PBAs are able to reversibly uptake Na ions, which render them as promising cathodes for SIBs. To this end, the HE‐PBA and ME‐PBA materials were subjected to galvanostatic cycling in the voltage range between 2.0 and 4.2 V versus Na^+^/Na (see **Figure** **3**a,b). The HE‐PBA electrode delivered a first‐cycle specific discharge capacity of about 120 mAh g^−1^ (calculated based on active material mass) at 0.01 A g^−1^, which is close to the theoretical specific capacity of approx. 140 mAh g^−1^. However, when one of the metal cations at the M position was removed, all ME‐PBAs revealed a slight decrease in specific capacity, and the removal of Fe resulted in the largest drop to 110 mAh g^−1^. Improved electrochemical performance of HE‐PBA was also observed on subsequent cycles. Upon increasing the specific current to 0.1 A g^−1^, HE‐PBA achieved initial specific capacities of about 100 mAh g^−1^, with enhanced cycling stability when compared with the ME‐PBAs. Note that the subtraction of Fe from the HE‐PBA did not alter the capacity retention, i.e., ME‐PBA(‐Fe) showed similar long‐term cycling performance to HE‐PBA, however, with 13% capacity lost from the beginning (100 vs 87 mAh g^−1^). Additionally, HE‐PBA clearly outperformed the ME‐PBAs. At the 150th cycle, for instance, the capacity retention was 94% for HE‐PBA, 89% for ME‐PBA(‐Mn), 88% for ME‐PBA(‐Co), 87% for ME‐PBA(‐Cu), and only 82% for ME‐PBA(‐Ni), suggesting that altering the Δ*S*_conf_ of the system has a strong influence on cycling stability. Also, the difference in capacity retention among the ME‐PBAs indicates that certain elements can help tailor the cyclability (e.g., Ni seems to play the most crucial role in the stability). This is also evident from the Coulombic efficiency (Figure 3a), following the trend of ME‐PBA(‐Ni) < ME‐PBA(‐Co) < ME‐PBA(‐Mn) < ME‐PBA(‐Fe) < ME‐PBA(‐Cu) < HE‐PBA. When comparing the electrochemical performance to conventional Fe‐PBA, the advantage is even more apparent, as the Fe‐PBA material showed relatively fast fading capacity with successive cycles, further confirming that increasing the Δ*S*_conf_ of the system significantly improves the cyclability (Figure S9a,b, Supporting Information).

A closer inspection of the voltage profiles (Figure 3b) revealed characteristic redox features at 3.45 and 3.22 V for charge and discharge, respectively, which is in accordance with the redox peaks from cyclic voltammograms (Figure 3c). More significant differences were observed when comparing the cyclic voltammetry results of HE‐PBA to conventional Fe‐PBA, see Figure S9c in the Supporting Information. Generally, for Fe‐PBA, two pairs of redox peaks at 2.95/2.65 and 3.65/3.27 V can be found, corresponding to the nitrogen‐coordinated high‐spin Fe^2+/3+^ and carbon‐coordinated low‐spin Fe^2+/3+^ redox couples, respectively.^[^ ^48^, ^49^ ^]^ It has been frequently reported that high‐spin Fe^2+/3+^ redox contributes strongly to the specific capacity,^[^ ^50^, ^51^ ^]^ while low‐spin Fe^2+/3+^ redox suffers from insufficient activation and poor reversibility,^[^ ^32^, ^52^, ^53^, ^54^ ^]^ consistent with the observation that the corresponding redox peaks decreased with cycling (Figure S9d, Supporting Information, marked with arrows). In contrast, for HE‐PBA, the low‐spin redox features were preserved, and remarkably, significantly increased the mean voltage. The redox peaks merged to become a single broad (asymmetric) peak, benefiting from the cocktail effects and providing high reversibility and cycling stability (note that the redox peak barely changed during the first couple of cycles, see Figure S9e, Supporting Information). Overall, HE‐PBA exhibited the highest mean discharge voltage [HE‐PBA (3.3 V) > ME‐PBA(‐Co) (3.26 V) > ME‐PBA(‐Mn) ≈ ME‐PBA(‐Ni) ≈ ME‐PBA(‐Cu) (3.25 V) > ME‐PBA(‐Fe) (3.19 V)]. Translating the capacity and voltage into gravimetric energy density assuming a theoretical sodium‐metal anode (Figure 3b), the largest specific energy of 378 Wh kg^−1^ was obtained for HE‐PBA, compared to 369 and 367 Wh kg^−1^ for ME‐PBA(‐Co) and ME‐PBA(‐Ni) and only 360, 354, and 344 Wh kg^−1^ for ME‐PBA(‐Mn), ME‐PBA(‐Cu), and ME‐PBA(‐Fe), respectively. HE‐PBA also showed the least voltage hysteresis of all materials tested in the present work. This agrees well with the cyclic voltammetry results shown in Figure 3c and further indicates that HE‐PBA has superior energy efficiency.

**Figure 4:**
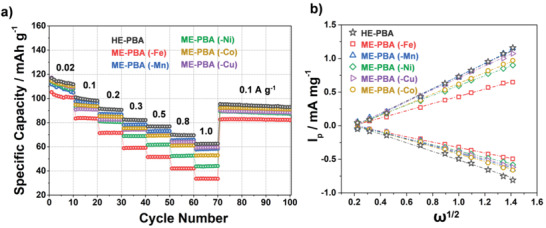
a) Multirate galvanostatic cycling of electrodes based on HE‐PBA (in black), ME‐PBA(‐Fe) (in red), ME‐PBA(‐Mn) (in blue), ME‐PBA(‐Ni) (in green), ME‐PBA(‐Co) (in dark yellow), or ME‐PBA(‐Cu) (in purple). Specific discharge capacities versus the cycle number are shown. b) The linear response of specific peak current as function of the square root of the sweep rate for determining the apparent sodium‐ion diffusion coefficient (see the Supporting Information for more details).

The rate capability of HE‐PBA and the different ME‐PBAs, as well as of Fe‐PBA, is presented in **Figure** **4**a and Figure S9b in the Supporting Information, with the corresponding voltage profiles shown in Figure S10 in the Supporting Information. Electrodes based on HE‐PBA delivered the largest specific discharge capacities at any of the specific currents, ranging from 0.02 to 1.0 A g^−1^. For instance, at 0.5 and 0.8 A g^−1^, HE‐PBA was still capable of achieving specific capacities of about 77 and 70 mAh g^−1^, respectively. For the ME‐PBAs, the rate capability was significantly reduced, especially for ME‐PBA(‐Fe), ME‐PBA(‐Ni), and ME‐PBA(‐Co). They delivered only about 33, 43, and 52 mAh g^−1^, respectively, at a high specific current of 1.0 A g^−1^, much lower than the corresponding discharge capacity of HE‐PBA (62 mAh g^−1^). Moreover, when decreasing the specific current to 0.1 A g^−1^ after high‐rate testing, the HE‐PBA electrode achieved 96 mAh g^−1^, outperforming all reference materials and emphasizing again the excellent reversibility of the de/sodiation reactions in HE‐PBA. When comparing with Fe‐PBA, the superiority of HE‐PBA is even more apparent (Figure S9b, Supporting Information). Besides, comparisons of the Na‐storage properties with recently reported single‐ (Δ*S*_conf_ = 0R) and dual‐metal PBAs (Δ*S*_conf_ < 1R) are shown in Tables S3 and S4 in the Supporting Information, highlighting the electrochemical performance of HE‐PBA in terms of cycling stability and rate capability.

To better understand the rate performance of the HE‐ and ME‐PBAs, the de/sodiation kinetics were studied in some more detail. The apparent sodium‐ion diffusion coefficient ($D_{{\text{Na}}^{+}}$) was calculated by performing cyclic voltammetry measurements with steadily increasing sweep rates (Figure S11, Supporting Information). All samples revealed a similar behavior, showing increasing peak separation and current with increasing sweep rate. Using the Randles–Sevcik equation (Equation (S3), Supporting Information), plotting the peak current as function of the square root of the sweep rate allows for the calculation of $D_{Na^{+}}$ (Figure 4b). More discussion is provided in the Supporting Information and the corresponding values are summarized in Table (S5) in the Supporting Information. HE‐PBA‐based electrodes exhibited the largest apparent sodium‐ion diffusion coefficients of 2.07 × 10^−8^ and 9.75 × 10^−9^ cm^2^ s^−1^ for desodiation and resodiation, respectively. Considering all materials have a similar structure and the electrodes were prepared under the same conditions, the de/sodiation kinetics follows the general trend of HE‐PBA > ME‐PBA (–Mn) > ME‐PBA (–Cu) > ME‐PBA (–Co) > ME‐PBA (–Ni) > ME‐PBA (–Fe) (based on the anodic trace [desodiation], Figure 4b), which agrees with the results from rate performance testing. Overall, this further confirms the improved kinetics for charge transfer achieved through the high‐entropy approach.

**Figure 5:**
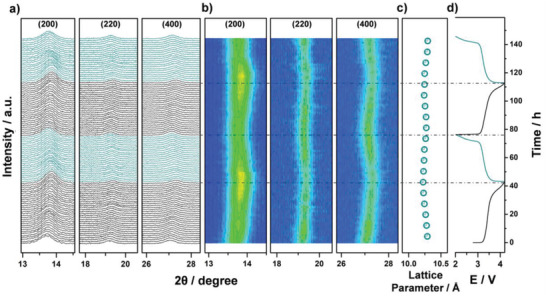
Operando XRD analysis of the electrochemical extraction/insertion of Na ions from/into HE‐PBA. a) Waterfall diagrams, b) contour plots of consecutively recorded patterns, c) lattice parameter changes, and d) the corresponding dis/charge curves for the first two cycles.

To resolve the relationship between structure and electrochemistry in this system, operando XRD was collected during two consecutive cycles of HE‐PBA against Na^+^/Na. A low specific current of 3.5 mA g^−1^ was applied to ensure sufficient sodium de/insertion and allow achieving a reasonable time resolution between XRD scans. The reflections from the HE‐PBA material only showed small variations during cycling, see **Figure** **5**a. Closely examining the (200), (220), and (400) reflections revealed a continuous but minor shift to larger 2θ values upon desodiation in the 1st cycle, corresponding to a contraction of the unit‐cell volume, accompanied by some peak broadening. This was most evident for the (200) reflection, which also exhibited an increase in intensity at the fully desodiated state (Figure 5b). In the subsequent discharge cycle, the reflections shifted back to their original 2θ values, with slightly lower intensity than in the initial pattern at the fully sodiated state. Similar behavior was found for the second cycle, implying the de/sodiation reactions are highly reversible. In fact, no phase transition was observed during the whole measurement, demonstrating that the extraction/insertion of Na ions from/into the HE‐PBA takes place via a solid‐solution (single‐phase) mechanism.^[^ ^55^, ^56^ ^]^ Figure 5c shows the changes of the cubic lattice parameter. As can be seen, the values remained in a narrow range of 10.23–10.31 Å, indicating the cathode material enables a nearly zero‐strain operation upon electrochemical cycling.

Taken together, HE‐PBA clearly has a more robust structure than the latest reported conventional single‐ or dual‐metal PBAs, as most of them undergo one or more phase transitions during de/sodiation (Table S6, Supporting Information). This result helps explain the superior cycling stability of HE‐PBA and further highlights the effectiveness of the high‐entropy strategy.

**Figure 6:**
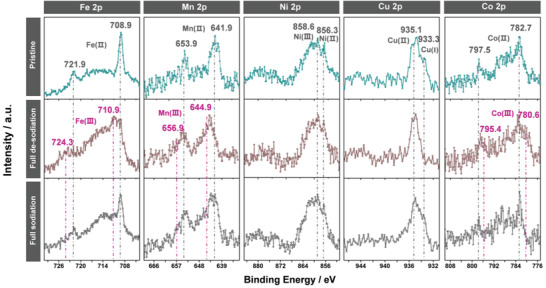
Ex situ XPS analysis of HE‐PBA‐based electrodes before/after cycling. Comparison of the Fe 2p, Mn 2p, Ni 2p, Cu 2p, and Co 2p core‐level spectra for the material in the pristine (in cyan), fully desodiated (charged to 4.2 V, in brown), and resodiated (discharged to 2.0 V, in black) states.

To better understand the role of each metal component in HE‐PBA, ex situ XPS analysis was conducted on electrodes before cycling and after the first charge and discharge (**Figure** **6**). As expected, for the pristine electrode, the Fe, Mn, Cu, and Co cations predominantly remained in the divalent state, but the peak intensities were lower compared with those for the HE‐PBA powder. The Ni 2p data (pristine state) revealed a larger fraction of trivalent Ni (858.6 eV) due to surface oxidation of Ni^2+^ during electrode preparation at ambient conditions (despite strong overlapping with the satellite peak).^[^ ^44^ ^]^ The Fe 2p, Mn 2p, and Co 2p spectra showed clear signs of partial oxidation of Fe^2+^ to Fe^3+^ (710.9 eV),^[^ ^47^, ^57^ ^]^ Mn^2+^ to Mn^3+^ (644.9 eV),^[^ ^39^ ^]^ and Co^2+^ to Co^3+^ (780.6 eV)^[^ ^45^, ^58^ ^]^ after the charge cycle (desodiation). Interestingly, Cu existed in a purely divalent state (935.1 eV), indicating that Cu^1+^ is getting oxidized to Cu^2+^ upon charging. After the discharge cycle (resodiation), the Fe 2p, Mn 2p, Co 2p, and Cu 2p data showed the reverse trend, i.e., the trivalent Fe, Mn, and Co cations were reduced to their divalent state and Cu^1+^ reappeared. In contrast, the Ni 2p peaks remained consistent after desodiation and resodiation, suggesting the Ni cations are redox inactive. Based on the above discussion, we conclude that Fe^2+/3+^, Mn^2+/3+^, Co^2+/3+^, and Cu^1+/2+^ are the main redox centers in HE‐PBA, with the Fe^2+/3+^ couple showing the greatest activity. These findings are in good agreement with the data shown in Figure 3. Regarding the Ni cations, despite being apparently redox inactive, their presence is beneficial to the cycling stability. Similar observations have been made for conventional FeNi‐PBAs.^[^ ^53^, ^59^, ^60^ ^]^

**Figure 7:**
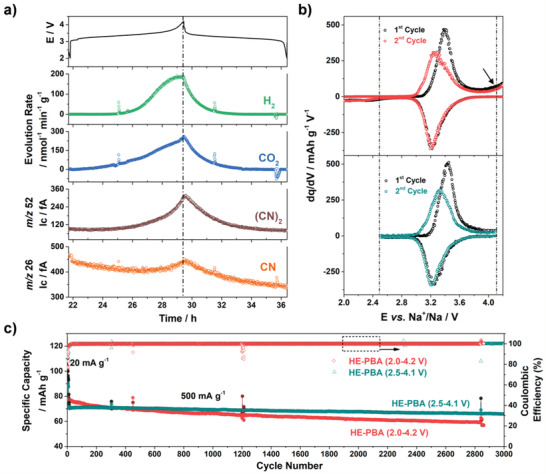
a) Gassing behavior of the HE‐PBA cathode during de/sodiation in the 2nd cycle from in situ DEMS. Evolution rates of H_2_ (green) and CO_2_ (blue) and ion currents for *m*/*z* = 52 (brown) and 26 (orange) are shown. b,c) Comparison of the cyclability of HE‐PBA in different voltage ranges: 2.0–4.2 V (in red/black) and 2.5–4.1 V (in cyan/black). b) Differential capacity plots of the first two cycles. c) Long‐term cycling performance at 0.5 A g^−1^ after 5 cycles at 0.02 A g^−1^.

Finally, the gassing behavior of the HE‐PBA cathode was probed in situ via differential electrochemical mass spectrometry (DEMS). To the best of our knowledge, such characterization has yet to be reported for PBA materials. To this end, the HE‐PBA‐based electrodes were subjected to galvanostatic cycling at 0.01 A g^−1^ using a customized DEMS cell setup, allowing the selective monitoring of gas evolution during electrochemical cycling. **Figure** **7**a shows the correlation between the voltage profile of the second cycle and the gas evolution rates for the most prominent gases, hydrogen (H_2_, *m*/*z* = 2) and carbon dioxide (CO_2_, *m*/*z* = 44). The full testing and the corresponding calibration curves are presented in Figure S12 in the Supporting Information. H_2_ evolution most likely stems from the reduction of crystal water and electrolyte reduction at the anode side, while CO_2_ was formed from oxidation of the organic carbonate electrolyte at the cathode.^[^ ^61^ ^]^ In general, the gas evolution did not change with the active material loading and was found to be about 80 and 33 µmol g^−1^ for H_2_ in the first and second cycle, respectively, amounting to a total of 113 µmol g^−1^. For CO_2_, about 73 and 56 µmol g^−1^ evolved, respectively, corresponding to 129 µmol g^−1^ in total. Figure 7a also shows a prominent signal for *m*/*z* = 52, particularly at high voltages, which can be explained by the oxidation of cyanide ligands of the HE‐PBA framework to dimeric cyanogen [(CN)_2_]. Further evidence for this is found in the *m*/*z* = 26 signal, corresponding to the CN monomer fragment. Oxidative dimerization of the host material anions is an apparent parallel to the well‐known release of oxygen from layered lithium metal oxide cathodes in LIBs.^[^ ^62^, ^63^, ^64^ ^]^ Unfortunately, quantification was not possible (no calibration standard available). Note that no signs of hydrogen cyanide (*m*/*z* = 27) evolution were observed. The evolution of (CN)_2_ is known to occur during thermal decomposition of certain metal hexacyanoferrates in air and under inert atmosphere,^[^ ^65^, ^66^ ^]^ while during battery operation, to the best of our knowledge, it is the first report of this kind of degradation of PBA cathodes. The irreversibility of such side reaction is evident at voltages above 4.1 V from the differential capacity plots in Figure 7b (marked by an arrow). In fact, literature review revealed that this phenomenon indeed exists in conventional PBAs;^[^ ^49^, ^67^ ^]^ however, it has not been discussed in detail so far. Nevertheless, further studies are clearly necessary to clarify the nature of degradation (mechanism, role of vacancies, etc.). Still, by narrowing the voltage window (2.5–4.1 V instead of 2.0–4.2 V), the side reactions were largely suppressed (Figure 7b), leading to further improvements in cycling stability, with a specific discharge capacity of 68 mAh g^−1^ after 3000 cycles, even when applying a high specific current of 0.5 A g^−1^ (Figure 7c).

## 3 Conclusion

We have demonstrated that high‐entropy versions of PBA cathode materials may show substantially enhanced electrochemical performance for sodium storage. Activating the reversibility of redox centers provides improvements in specific capacity and capacity retention by ensuring better structural stability during cycling. Increased configurational entropy of the system was achieved by introducing five metal cations at equimolar fractions at the same nitrogen‐coordinated lattice site. Such a structure is reported for the first time and enables a nearly zero‐strain operation upon sodium de/insertion. Moreover, side reactions, potentially leading to the degradation of PBA‐based electrode materials (especially at high voltages), have been discussed in the context of gas evolution. Overall, applying the high‐entropy strategy to MOFs might pave the way toward development of stable and low‐cost host materials for various secondary battery systems.

## Conflict of Interest

The authors declare no conflict of interest.

## Supporting information

Supporting Information

## Data Availability

The data that support the findings of this study are available from the corresponding author upon reasonable request.
