# Supplementary material for: High‐Entropy Metal–Organic Frameworks for Highly Reversible Sodium Storage
Source: Adv Mater. 2021 Jul 9;33(34):2101342. doi: 10.1002/adma.202101342 (PMC11469271; doi:10.1002/adma.202101342)
Supplement: Supplementary file 1 — Supporting Information [file ADMA-33-2101342-s001.pdf]

© 2021 Wiley-VCH GmbH

ADVANCED MATERIALS

Supporting Information

for *Adv. Mater.,* DOI: 10.1002/adma.202101342

High-Entropy Metal–Organic Frameworks for Highly Reversible Sodium Storage

Yanjiao Ma, Yuan Ma, Sören Lukas Dreyer, Qingsong Wang, Kai Wang, Damian Goonetilleke, Ahmad Omar, Daria Mikhailova, Horst Hahn, Ben Breitung,* and Torsten Brezesinski**

Supporting Information

High-Entropy Metal-Organic Frameworks for Highly Reversible Sodium Storage

Yanjiao Ma,[a] Yuan Ma,[a,]* Sören Lukas Dreyer,[a] Qingsong Wang,[a] Kai Wang,[a] Damian Goonetilleke,[a] Ahmad Omar,[b] Daria Mikhailova,[b] Horst Hahn,[a,c,d] Ben Breitung[a,]* and Torsten Brezesinski[a,]*

[a] *Institute of Nanotechnology, Karlsruhe Institute of Technology (KIT), Hermann-von-Helmholtz Platz 1, 76344 Eggenstein-Leopoldshafen, Germany*

[b] *Leibniz Institute for Solid State and Materials Research (IFW) Dresden, Helmholtzstr. 20, 01069 Dresden, Germany*

[c] *Joint Research Laboratory Nanomaterials – Technische Universität Darmstadt and Karlsruhe Institute of Technology (KIT), Otto-Berndt-Str. 3, 64206 Darmstadt, Germany*

[d] *Helmholtz Institute Ulm (HIU) for Electrochemical Energy Storage, Helmholtzstr. 11, 89081 Ulm, Germany*

*Corresponding authors: yuan.ma@kit.edu (Yu.M.), ben.breitung@kit.edu (B.B.), torsten.brezesinski@kit.edu (T.B.)

Methods

Materials Synthesis. High-entropy Prussian blue analogue $Na_x(FeMnNiCuCo)[Fe(CN)_6]$ (HE-PBA) was synthesized by co-precipitation at room temperature. Specifically, 4 mmol $Na_4Fe(CN)_6$ and 15 g NaCl were dissolved in 200 mL deionized water to form solution A. 2.5 mmol metal precursors ($FeCl_2 \cdot 4H_2O$, $MnCl_2 \cdot 4H_2O$, $NiCl_2 \cdot 6H_2O$, $CuCl_2 \cdot 2.5H_2O$ and $CoCl_2 \cdot 6H_2O$, each 0.5 mmol) were dissolved in 200 mL deionized water to form solution B. Solutions A and B were slowly and simultaneously dropwise added to 500 mL deionized water under constant magnetic stirring to form solution C. After stirring for 2 h, solution C was further aged at room temperature for 24 h. The precipitate was collected by centrifugation, washed several times with deionized water and eventually dried at 60 °C overnight. The medium-entropy $Na_x(MnNiCoCu)[Fe(CN)_6]$ (ME-PBA(-Fe)), $Na_x(FeNiCoCu)[Fe(CN)_6]$ (ME-PBA(-Mn), $Na_x(FeMnCoCu)[Fe(CN)_6]$ (ME-PBA(-Ni)), $Na_x(FeMnNiCu)[Fe(CN)_6]$ (ME-PBA(-Co)), $Na_x(FeMnNiCo)[Fe(CN)_6]$ (ME-PBA(-Cu) and conventional $Na_xFe[Fe(CN)_6]$ were prepared analogously by using the respective metal precursors.

Characterization. The crystal structure of the samples was investigated *via* powder X-ray diffraction (XRD) using either a Bruker D8 Advance (Cu-$K_{\alpha 1}$ radiation, λ = 1.54056 Å) or a STOE Stadi P diffractometer equipped with a Ga-jet X-ray source (Ga-K_β radiation, λ = 1.20793 Å). X-ray absorption spectroscopy (XAS) was carried out at the beamline P65 of PETRA III extension of DESY in Hamburg, Germany. The measurements were performed on powder sample sealed in Kapton tape in transmission mode for all edges. Metal foils as standards were measured at the same time. XAS data was calibrated and analyzed using the Athena program.[1] For the extended X-ray absorption fine structure (EXAFS), a linear fit of the pre-edge was performed and a third-order polynomial fit was used to describe the post-edge background. Attenuated total reflection-infrared (ATR-IR) spectroscopy was performed on an ALPHA FT-IR spectrometer (Bruker) in an argon glovebox. The spectra were collected using the OPUS software. Thermogravimetric analysis (TGA, TA Instruments Q5000) was performed at a heating rate of 5 °C min^{-1} under ambient air. The sample morphology

was characterized by means of scanning electron microscopy (SEM, Zeiss LEO 1530) and transmission electron microscopy (TEM). High-angle annular dark-field (HAADF) scanning TEM (STEM) was conducted at 300 kV using a Thermo Fisher Themis Z equipped with a probe-corrector (S-CORR). Drift-corrected elemental mapping was done using an energy-dispersive X-ray spectroscopy (EDS) detector (Thermo Fisher Super-X). Inductively coupled plasma-optical emission spectroscopy (ICP-OES) was performed on a SPECTRO ARCOS SOP instrument. For ICP-OES analysis, the samples were dissolved in hydrochloric acid and diluted with deionized water prior to the measurement. X-ray photoelectron spectroscopy (XPS) measurements were carried out on a PHI 5800 MultiTechnique ESCA system (Physical Electronics). The spectra were acquired at a take-off angle of 45° using monochromatic Al-K_α radiation (λ = 1486.6 eV) and pass energies at the electron analyzer of 29.35 and 93.9 eV for detail and survey scans, respectively. The C1s peak was used for binding energy calibration and set to 284.8 eV for adventitious carbon.

Electrode Preparation. First, all as-prepared samples were pre-dried at 120 °C for two days to remove trace water. The HE-PBA- and ME-PBA-based electrodes were composed of active material (70 wt.%), Super C65 carbon black additive (TIMCAL, 20 wt.%) and polyvinylidene difluoride binder (PVdF 5130, Solvay, 10 wt.%). For the slurry preparation, PVdF was dissolved in *N*-methyl-2-pyrrolidone (Sigma-Aldrich) and subsequently Super C65 and active material were added. The resulting mixture was dispersed using a Thinky ARE-250 mixer (two times, each for 3 min). Then, the electrode paste was cast onto aluminum foil using a laboratory doctor blade (180 µm wet-film thickness). After drying in a vacuum at 120 °C for 24 h, circular electrodes (12 mm in diameter) were punched out and further dried for 10 h. The areal active material loading was in the range between 2.0 and 2.5 mg cm^{-2}.

Electrochemical Testing. The electrochemical performance was evaluated either in CR2032 coin cells or in Swagelok-type three-electrode cells using sodium metal as counter and reference electrodes. All cells were assembled in an argon glovebox (MBraun UNIlab, [H_2O] and [O_2] < 0.1 ppm). The electrolyte consisted of a 1 M solution of $NaClO_4$ in ethylene carbonate/dimethyl

carbonate/propylene carbonate (EC:DMC:PC, 1:1:1 by volume) with 5 % fluoroethylene carbonate (FEC). Prior to electrochemical characterization, the cells were allowed to rest for 6 h. Cyclic voltammetry was carried out in the voltage range of 2.0-4.2 V vs. Na^+/Na using a VMP3 potentiostat (BioLogic). Galvanostatic cycling tests were performed by means of a battery tester (MACCOR model 4300) with cut-off voltages of 2.0 and 4.2 V vs. Na^+/Na. All electrochemical measurements were performed in climatic chambers with a set temperature of (20 ± 1) °C.

***Operando* XRD Analysis.** *Operando* XRD measurements were carried out using a customized coin cell with Kapton windows. The electrodes were the same as those used for the electrochemical testing, but with a higher areal active material loading of around 5 mg cm^{-2}. Galvanostatic cycling was performed at a specific current of 3.5 mA g^{-1} using a potentiostat/galvanostat (SP-150, BioLogic) with cut-off voltages of 2.0 and 4.2 V vs. Na^+/Na.

***Ex Situ* XPS Analysis.** For *ex situ* XPS characterization of HE-PBA, the cells were subjected to one full charge and one full discharge cycle at 5 mA g^{-1} and subsequently opened in an argon glovebox. The electrodes were rinsed with DMC to remove residual electrolyte and then dried inside the glovebox. Finally, they were mounted in a sealed XPS holder to avoid air exposure.

***Operando* Gas Analysis.** Gas evolution of HE-PBA upon electrochemical cycling was probed by differential electrochemical mass spectrometry (DEMS). A custom cell made from a 30 mm-diameter cathode (5.4 mg cm^{-2} areal active material loading) with a 4 mm hole in the center to allow for gas flow, a 40 mm-diameter GF/D glass microfiber separator, a 32 mm-diameter sodium metal anode and 800 mL electrolyte (the same electrolyte as used in the coin cells) was cycled at 10 mA g^{-1} in the voltage range between 2.0 and 4.2 V vs. Na^+/Na. During the DEMS measurement, a constant stream of He carrier gas (purity 6.0, 2.5 mL min^{-1}) was passed through the cell. The extracted gas mixture was analyzed using a mass spectrometer (Omni Star GSD 320, Pfeiffer Vacuum GmbH). Further details can be found in the literature.[2,3]

Calculation of configurational entropy

For a random solid solution, the ideal configurational entropy per mole can be expressed as:[4]

$$\Delta S_{\text{conf}} = -R\sum_{i=1}^{n} x_i \ln x_i, \qquad \textbf{(Equation S1)}$$

where R is the ideal gas constant and x_i represents the molar fraction of the ith component. For a given number of components n, the configurational entropy reaches the largest value when the atomic fraction of all components is the same (i.e., equimolar). Then, the configurational entropy per mole is:[5]

$$\Delta S_{\text{conf}} = -R\left({}^{1}\!/_{n} \ln {}^{1}\!/_{n} + {}^{1}\!/_{n} \ln {}^{1}\!/_{n} + \cdots {}^{1}\!/_{n} \ln {}^{1}\!/_{n}\right) = -R \ln {}^{1}\!/_{n} = R \ln n. \qquad \textbf{(Equation S2)}$$

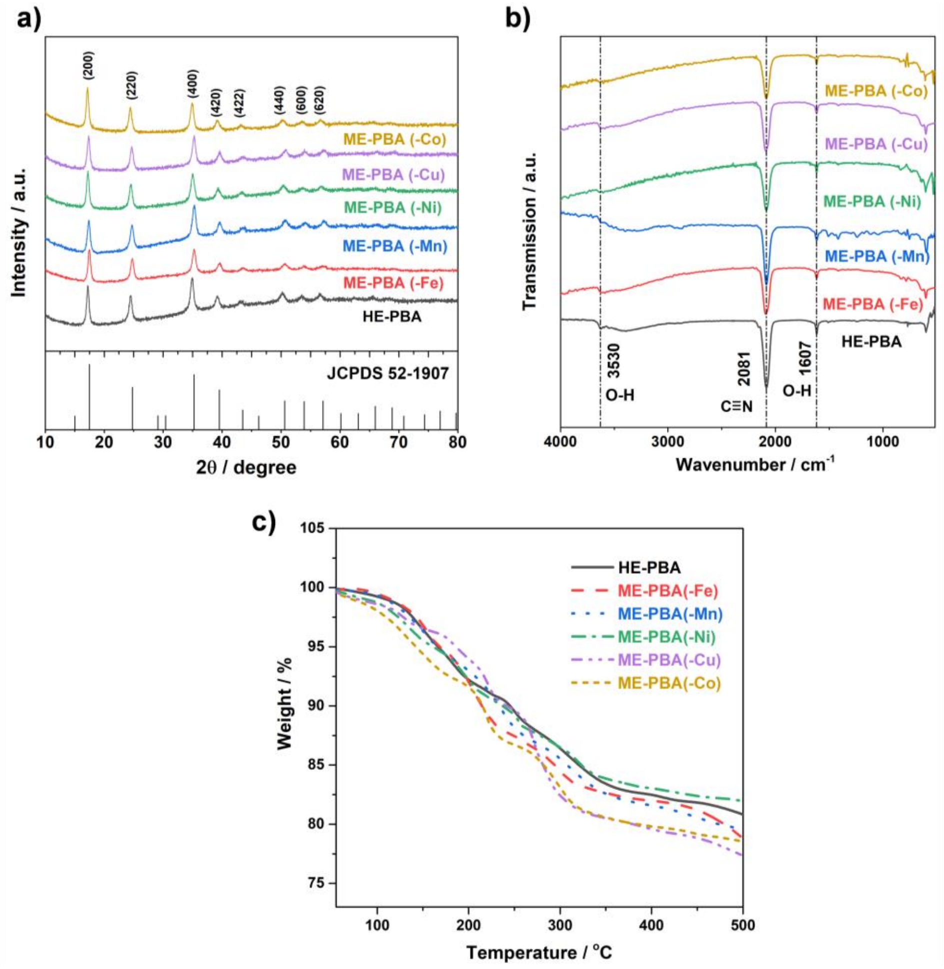

Figure S1. (a) XRD patterns, (b) ATR-IR spectra and (c) TGA curves for HE- and ME-PBAs.

Table S1. Structural parameters and disagreement indices of the conducted Rietveld refinement for the XRD pattern of HE-PBA.

Atom	Wyckoff Pos.	Atomic Position			Occ	ADP (U_{iso} / Å^2)
		x	*y*	*z*		
Fe1	4a	0	0	0	0.667(8)	0.012
Fe2	4b	0.5	0	0	0.2	0.0033(19)
Mn	4b	0.5	0	0	0.2	0.0033(19)
Ni	4b	0.5	0	0	0.2	0.0033(19)
Cu	4b	0.5	0	0	0.2	0.0033(19)
Co	4b	0.5	0	0	0.2	0.0033(19)
C	24e	0.2176(21)	0	0	0.667(8)	0.02
N	24e	0.2629(32)	0	0	0.667(8)	0.02

Na	8c	0.25	0.25	0.25	1	0.1113(41)
Space group = $Fm{-}3m$, $a = b = c$ = 10.2708(13) Å, V = 1083.45(40) Å^3, R_w = 1.48 %						

Rietveld refinement was used to determine structural parameters of the HE-PBA material. The reflections could be indexed to a cubic crystal symmetry with the space group $Fm{-}3m$. A vacancy-containing $Na_xFe[Fe(CN)_6]_{1-y}$ model (ICSD coll. code 193354) was refined against the diffraction data, and the corresponding Rietveld refinement profile and structural parameters are shown in **Figure 1c** and **Table S1**, respectively. The material was found to exhibit a cell volume of 1083.45 Å^3 with lattice parameters $a = b = c$ = 10.271(1) Å and α = β = γ = 90 °, which represents a contraction relative to the undoped material previously reported by Pramudita *et al.*[6] Although the substituted ions, Mn^{2+}, Ni^{2+}, Cu^{2+} and Co^{2+}, exhibit a larger ionic radius than Fe^{2+}, and hence cause lattice expansion, previous studies have shown that the substitution of divalent ions can decrease the ionic radius of the ferrocyanide ion, resulting in overall lattice contraction.[7] This is consistent with the refined atomic positions of the C and N sites in the structure, which exhibit a slight displacement from the positions reported for the pristine material. To model possible $[Fe(CN)_6]^{x-}$ vacancies, the occupancy of the Fe (4a), C (24e) and N (24e) sites were allowed to vary, but constrained to be equal. The formation of $[Fe(CN)_6]^{x-}$ in this system is typically associated with coordinated water content in the structure, which usually occupies the same region as the Na site.[8] This would also account for the relatively large atomic displacement parameter (ADP) determined for the Na site, as this parameter can show strong correlation with site occupancy factors. The refined occupancy of the Fe and coordinated C, N sites was found to be 0.667(8), suggesting the presence of $[Fe(CN)_6]^{x-}$ in this material. This occupancy of 0.667(8):1 between the $[Fe(CN)_6]^{x-}$ and Na site is also in good agreement with the chemical formula ratio of 0.79:1.19 (≈0.664:1) determined from ICP-OES, see **Table S2**.

Table S2. ICP-OES results of element ratios for HE-PBA, ME-PBA(-Fe), ME-PBA(-Mn), ME-PBA(-Ni), ME-PBA(-Cu) and ME-PBA(-Co).

	Na	Fe	Mn	Ni	Cu	Co
**HE-PBA**	1.193(2)	0.993(3)	0.203(3)	0.201(2)	0.199(3)	0.201(4)
	$Na_{1.19}Fe_{0.2}Mn_{0.2}Ni_{0.2}Cu_{0.2}Co_{0.2}[Fe(CN)_6]_{0.79}\square_{0.21}\cdot 1.16H_2O$					
**ME-PBA(-Fe)**	1.190(4)	0.782(3)	0.251(6)	0.250(3)	0.252(1)	0.250(2)
	$Na_{1.19}Mn_{0.25}Ni_{0.25}Cu_{0.25}Co_{0.25}[Fe(CN)_6]_{0.78}\square_{0.22}\cdot 1.19H_2O$					
**ME-PBA(-Mn)**	1.212(4)	1.042(3)	0	0.250(6)	0.249(5)	0.251(5)
	$Na_{1.21}Fe_{0.25}Ni_{0.25}Cu_{0.25}Co_{0.25}[Fe(CN)_6]_{0.79}\square_{0.21}\cdot 1.21H_2O$					
**ME-PBA(-Ni)**	1.201(4)	1.034(2)	0.250(2)	0	0.251(5)	0.250(4)
	$Na_{1.20}Fe_{0.25}Mn_{0.25}Cu_{0.25}Co_{0.25}[Fe(CN)_6]_{0.78}\square_{0.22}\cdot 1.14H_2O$					
**ME-PBA(-Cu)**	1.194(3)	1.052(2)	0.251(3)	0.253(1)	0	0.250(2)
	$Na_{1.19}Fe_{0.25}Mn_{0.25}Ni_{0.25}Co_{0.25}[Fe(CN)_6]_{0.80}\square_{0.20}\cdot 1.20H_2O$					
**ME-PBA(-Co)**	1.191(4)	1.041(2)	0.253(3)	0.249(3)	0.250(1)	0
	$Na_{1.19}Fe_{0.25}Mn_{0.25}Ni_{0.25}Cu_{0.25}[Fe(CN)_6]_{0.79}\square_{0.21}\cdot 1.16H_2O$					

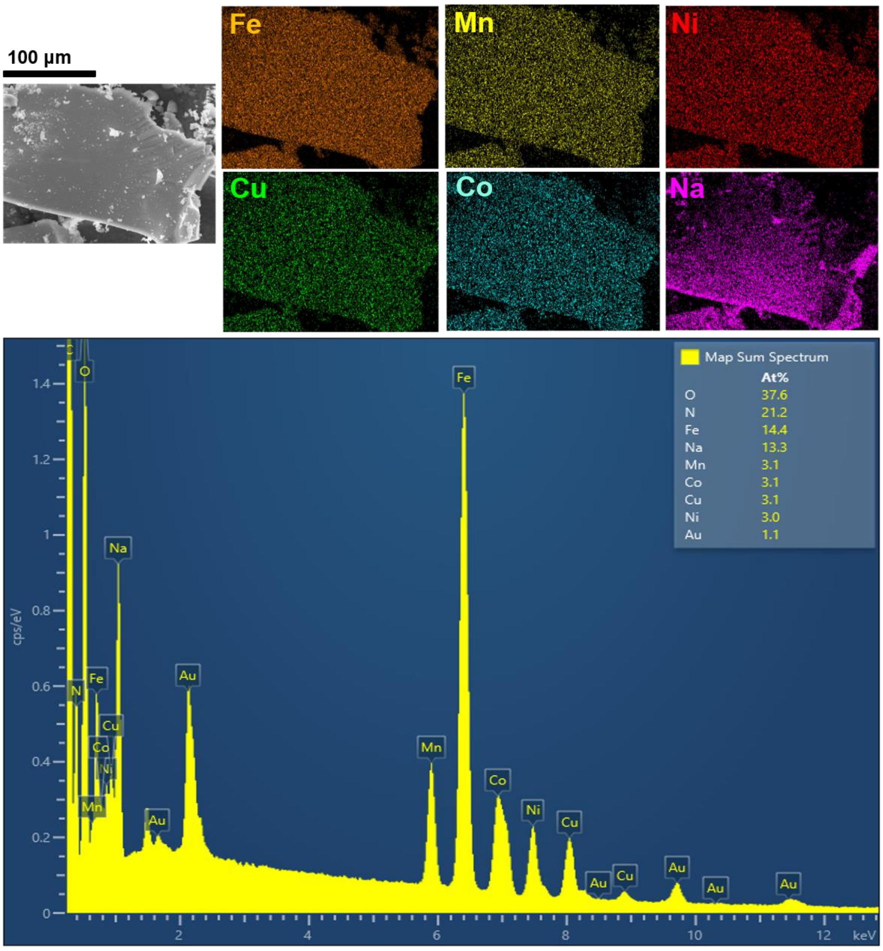

Figure S2. SEM micrograph and EDS mapping analysis for HE-PBA.

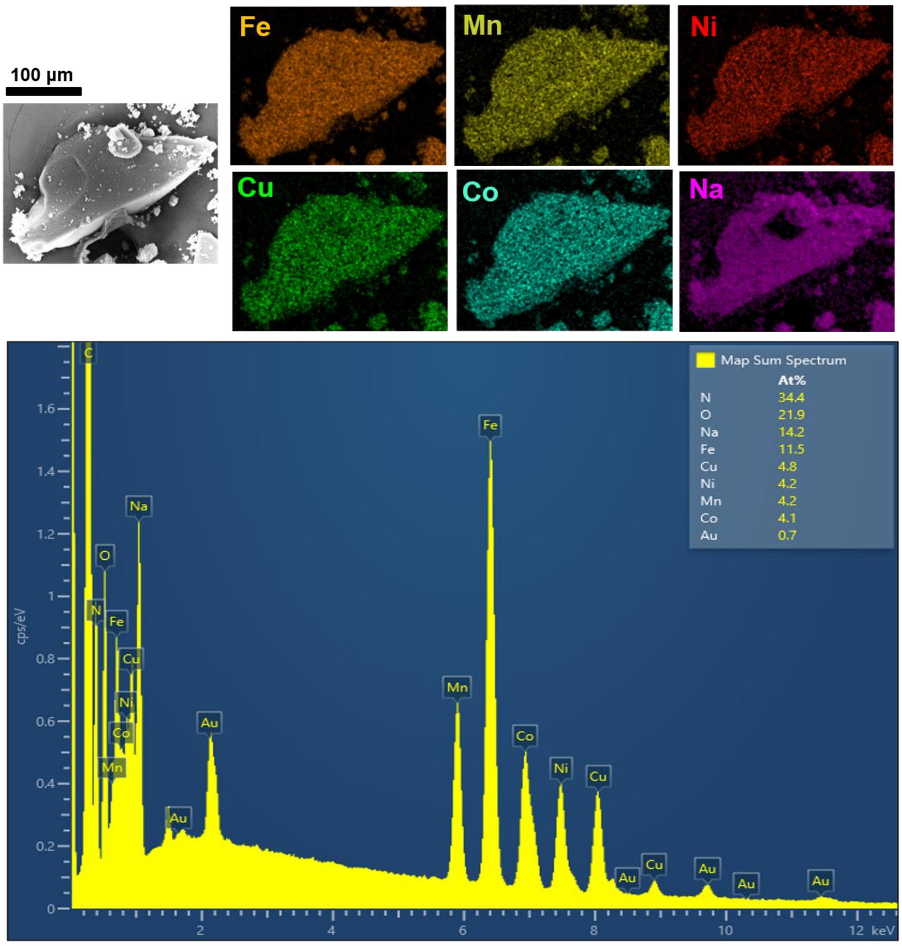

Figure S3. SEM micrograph and EDS mapping analysis for ME-PBA(-Fe).

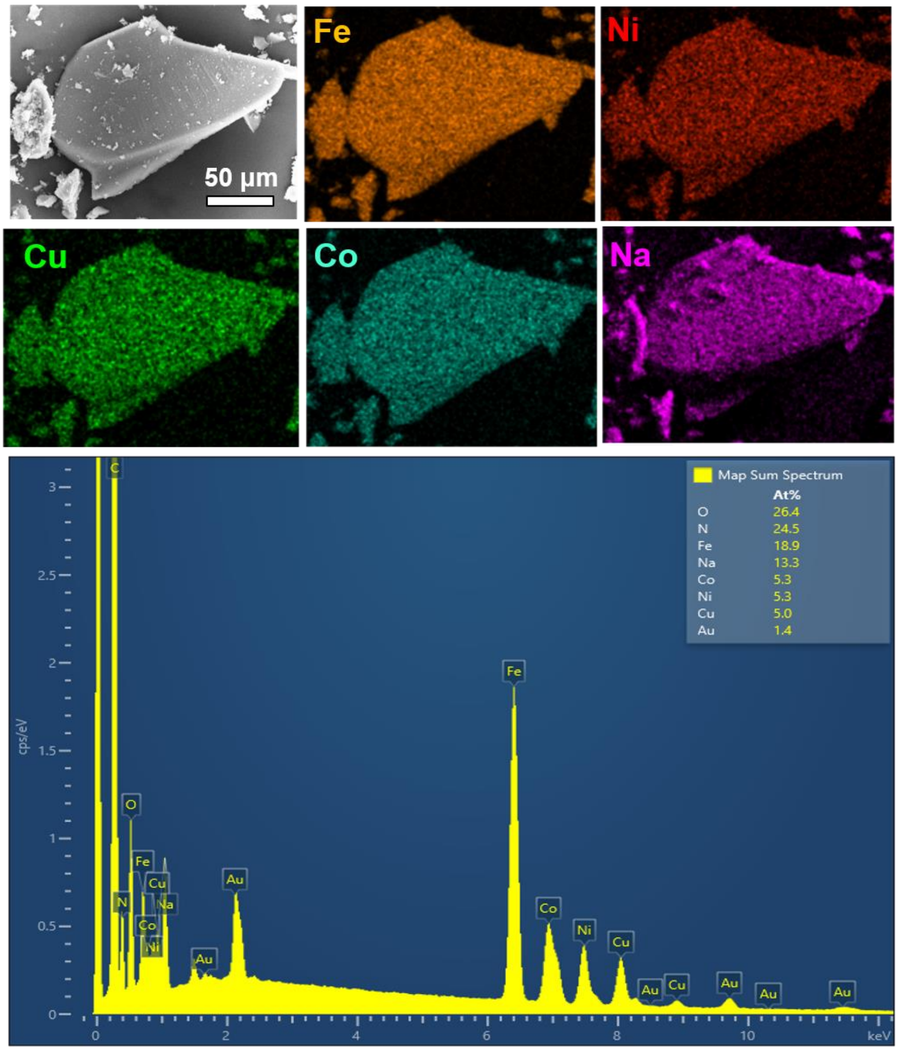

Figure S4. SEM micrograph and EDX mapping analysis for ME-PBA(-Mn).

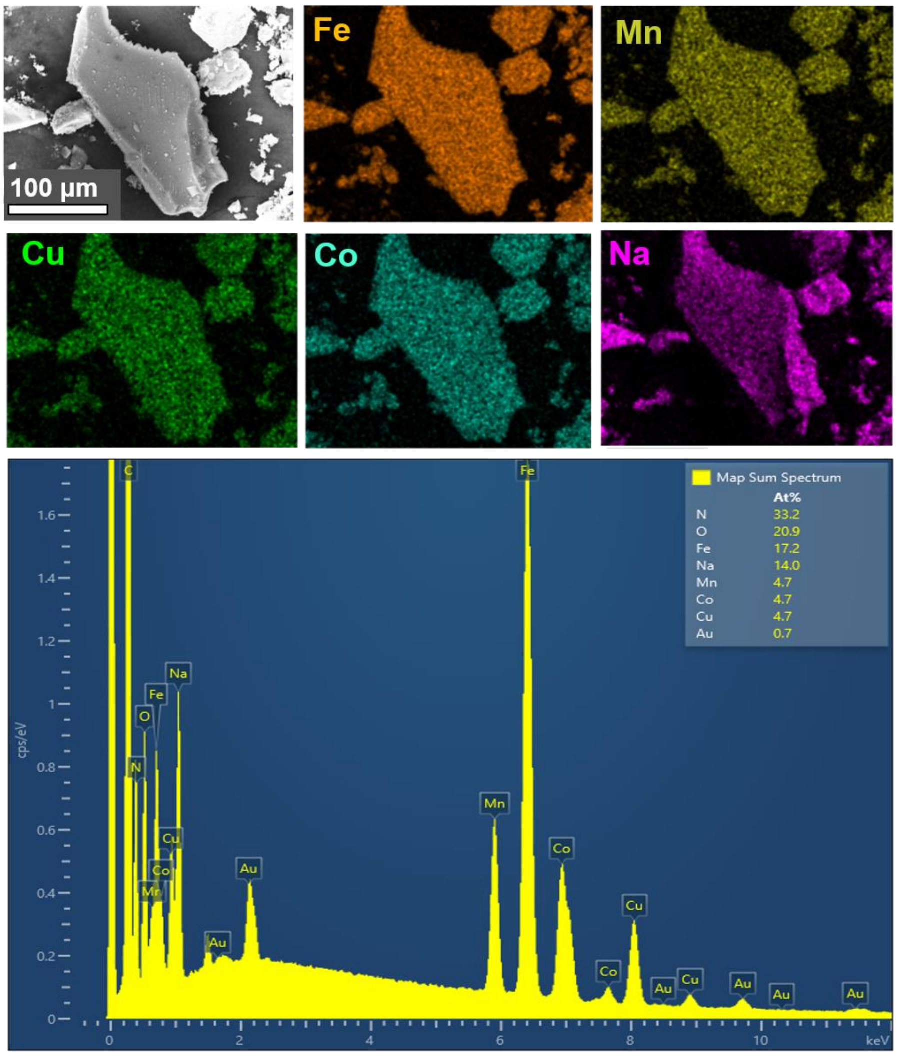

Figure S5. SEM micrograph and EDS mapping analysis for ME-PBA(-Ni).

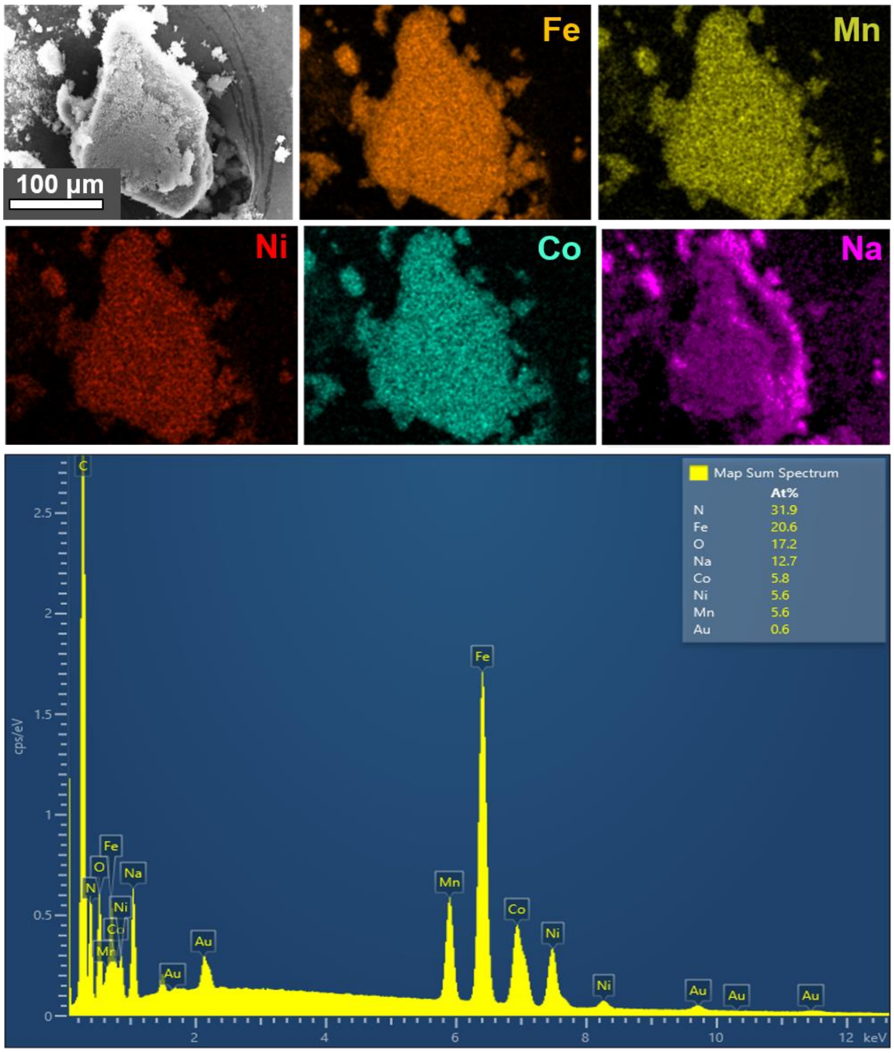

Figure S6. SEM micrograph and EDS mapping analysis for ME-PBA(-Cu).

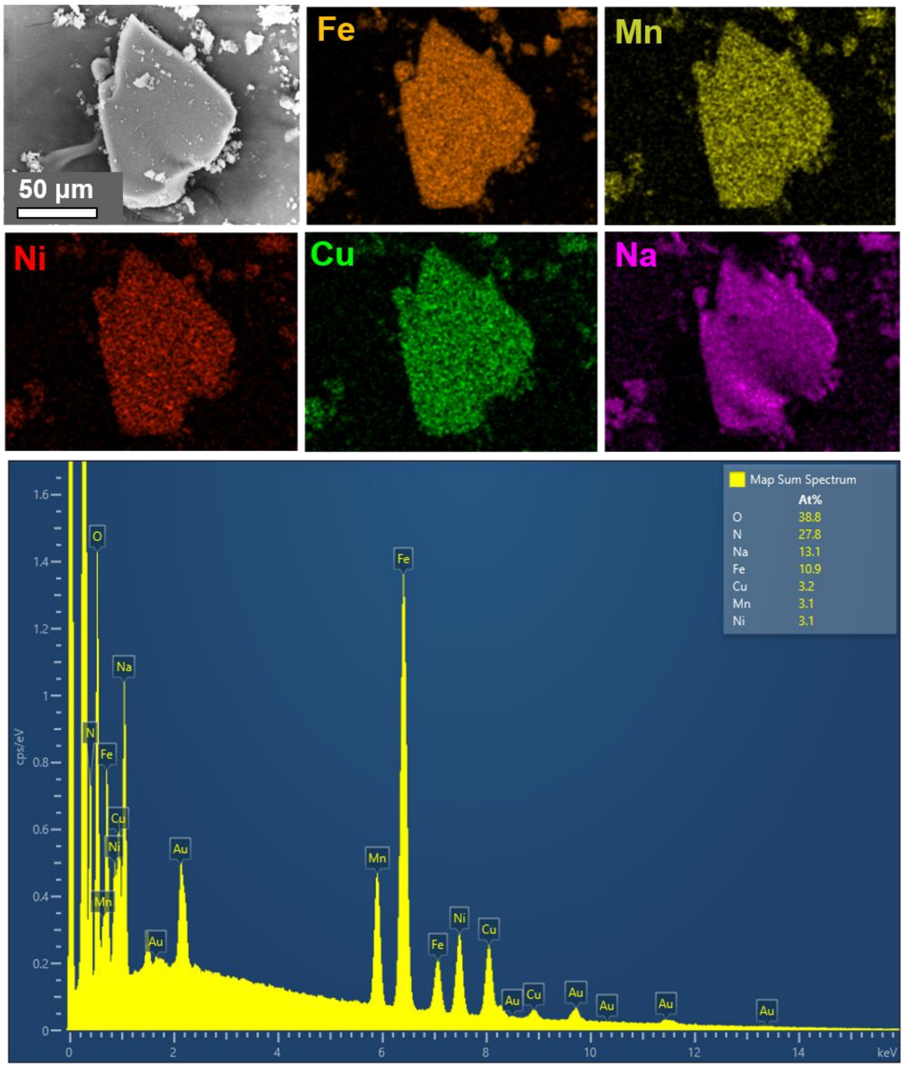

Figure S7. SEM micrograph and EDS mapping analysis for ME-PBA(-Co).

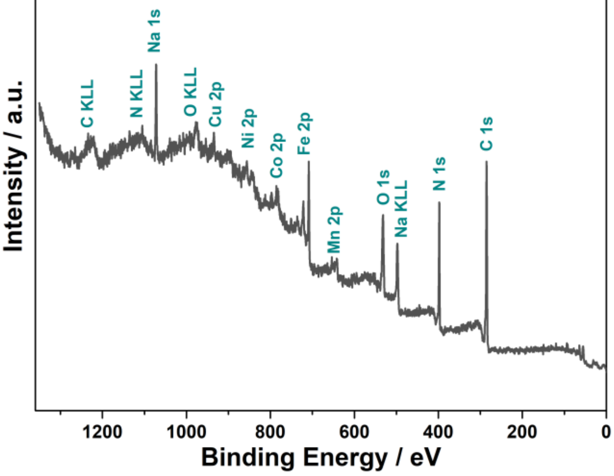

Figure S8. XP survey spectrum for HE-PBA.

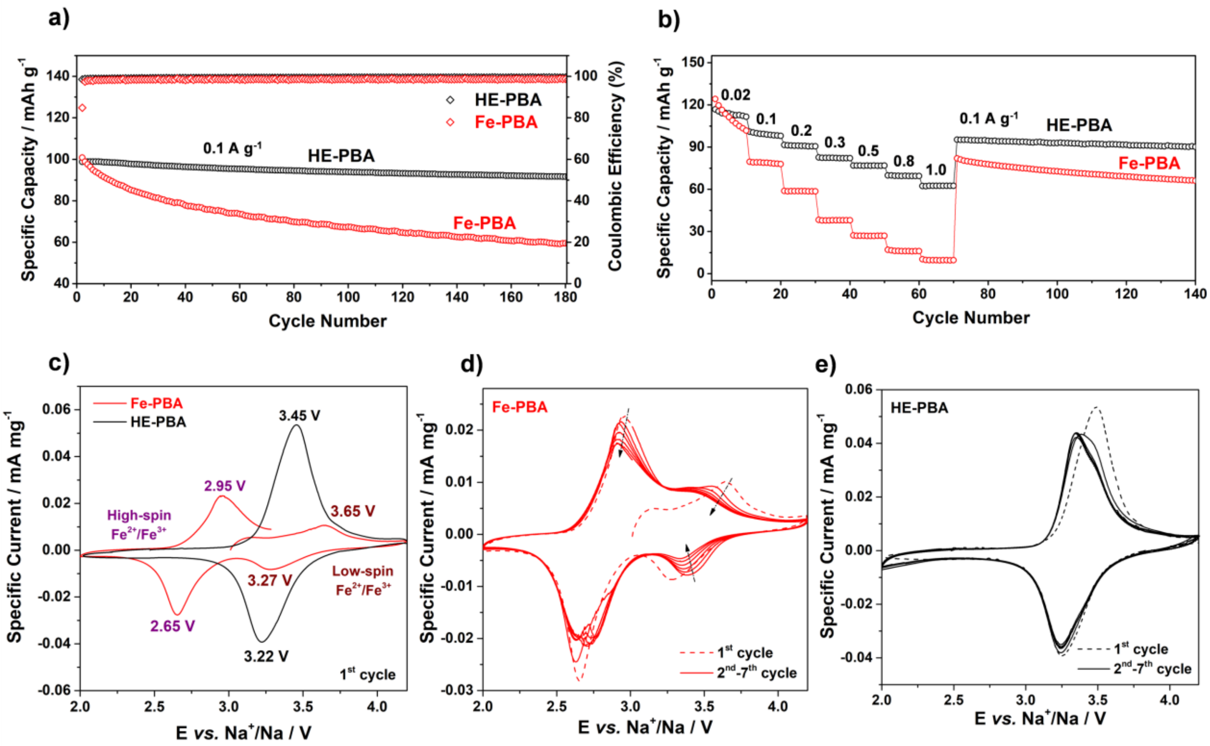

Figure S9. Comparison of the electrochemical performance of HE-PBA and conventional Fe-PBA. (a) Galvanostatic cycling at 0.1 A g^{-1} (1st cycle is omitted for clarity) and (b) rate capability. (c-e) Cyclic voltammograms for both materials at a sweep rate of 0.05 mV s^{-1}, (c) 1st cycle and (d, e) 1st to 7th cycles.

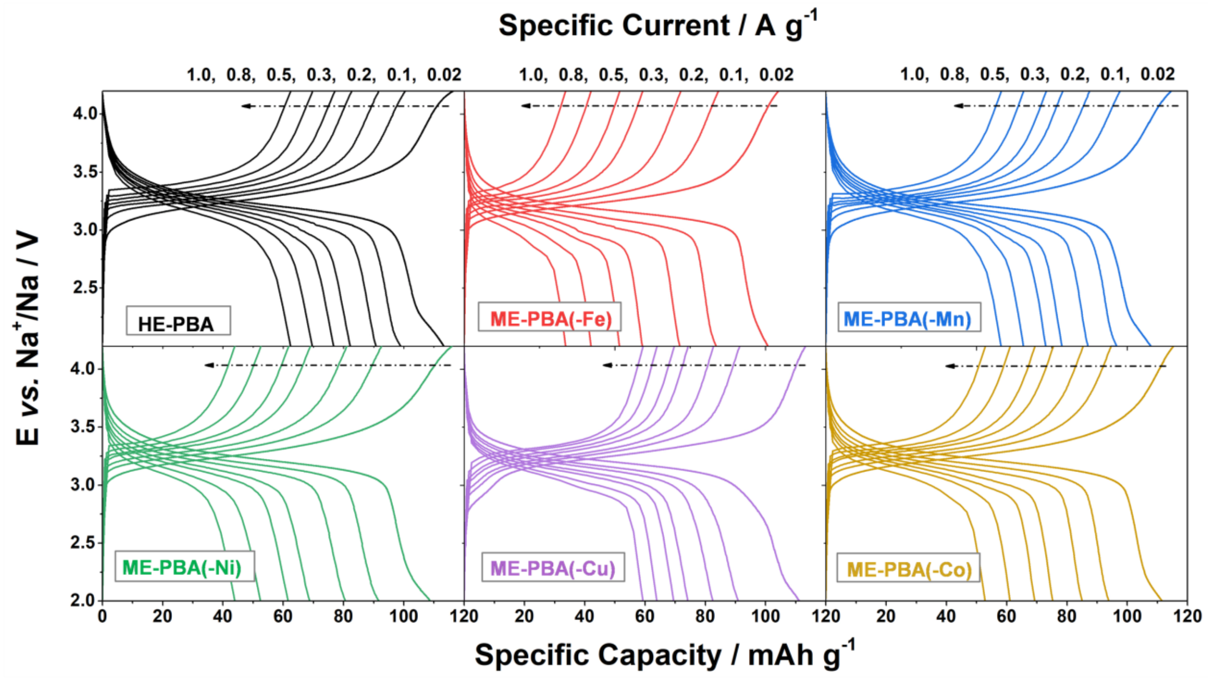

Figure S10. Voltage profiles for electrodes based on HE-PBA (in black), ME-PBA(-Fe) (in red), ME-PBA(-Mn) (in blue), ME-PBA(-Ni) (in green), ME-PBA(-Cu) (in purple) or ME-PBA(-Co) (in dark yellow). Every 5th cycle for each specific current rate is shown.

Table S3. Comparison of the (long-term) cycling performance of HE-PBA/ME-PBAs and conventional (single- or dual-metal) PBAs as cathode active materials in sodium-ion cells.

Material	Configurational Entropy	Specific Current (mA g^{-1})	Cycle No.	Specific Capacity (mAh g^{-1})	Ref.
**HE-PBA**	**1.61R**	**100**	**200**	**93**	This work
		**500**	**3000**	**68**	
ME-PBA(-Mn)	1.39R	100	200	88	
ME-PBA(-Co)		100	200	85	
ME-PBA(-Cu)		100	200	83	
ME-PBA(-Fe)		100	200	82	
ME-PBA(-Ni)		100	200	79	
$Na_xFeNi[Fe(CN)_6]$	< 1R	10	100	101	[9]
$Na_xFeNi[Fe(CN)_6]$		200	90	80	[8]
$Na_xMnNi[Fe(CN)_6]$		100	800	80	[10]
$Na_xFeCu[Fe(CN)_6]$		50	1000	72	[11]
$Na_xNiCo[Fe(CN)_6]$		50	100	82	[12]
$Na_xMnFe[Fe(CN)_6]$		50	300	80	[13]
$K_xNa_yMn[Fe(CN)_6]$		40	100	107	[14]
$Na_xFe[Fe(CN)_6]$	0R	50	150	97	[15]
$Na_xMn[Fe(CN)_6]$		6	30	120	[16]
$Na_{1+x}Fe[Fe(CN)_6]$		20	400	99	[17]
$Na_xNi[Fe(CN)_6]$		30	50	65	[18]
$Cu_3[Fe(CN)_6]_2$		20	100	20	[19]
$Na_xNi[Fe(CN)_6]$		50	100	57	[12]
$Na[Fe(CN)_6]$		85	1000	70	[20]
$K_xFe[Fe(CN)_6]$		10	30	62	[21]
$Na_xMn[Fe(CN)_6]$		200	500	80	[22]
$Na_xCo[Fe(CN)_6]$		50	100	60	[12]
$Na_xCo[Fe(CN)_6]$		30	50	86	[18]

Table S4. Comparison of the rate capability of HE/ME-PBAs and conventional (single- or dual-metal) PBAs as cathode active materials in sodium-ion cells.

Material	Configurational Entropy	Specific Current (mA g^{-1})	Specific Capacity (mAh g^{-1})	Ref.
**HE-PBA**	**1.61R**	**1000**	**62**	**This work**
ME-PBA(-Cu)	1.39R	1000	59	
ME-PBA(-Mn)		1000	58	
ME-PBA(-Co)		1000	52	
ME-PBA(-Ni)		1000	43	
ME-PBA(-Fe)		1000	33	
$Na_xFeNi[Fe(CN)_6]$	< 1R	500	50	[9]
$Na_xFeNi[Fe(CN)_6]$		1000	40	[8]
$Na_xMnFe[Fe(CN)_6]$		2000	21	[13]
$Na_xMnNi[Fe(CN)_6]$		600	50	[10]
$Na_xNiCo[Fe(CN)_6]$		750	63	[23]
$Na_xFeCu[Fe(CN)_6]$		800	57	[11]
$Na_xMn[Fe(CN)_6]$	0R	1500	22	[18]
$Na_xNi[Fe(CN)_6]$		800	50	[18]
$Na_xNi[Fe(CN)_6]$		800	34	[12]
$Mn_2[Fe(CN)6]$		1000	49	[24]
$Na_xMn[Fe(CN)_6]$		600	73	[22]
$Na_xFe[Fe(CN)_6]$		400	80	[25]
$Na_xCo[Fe(CN)_6]$		1000	30	[18]

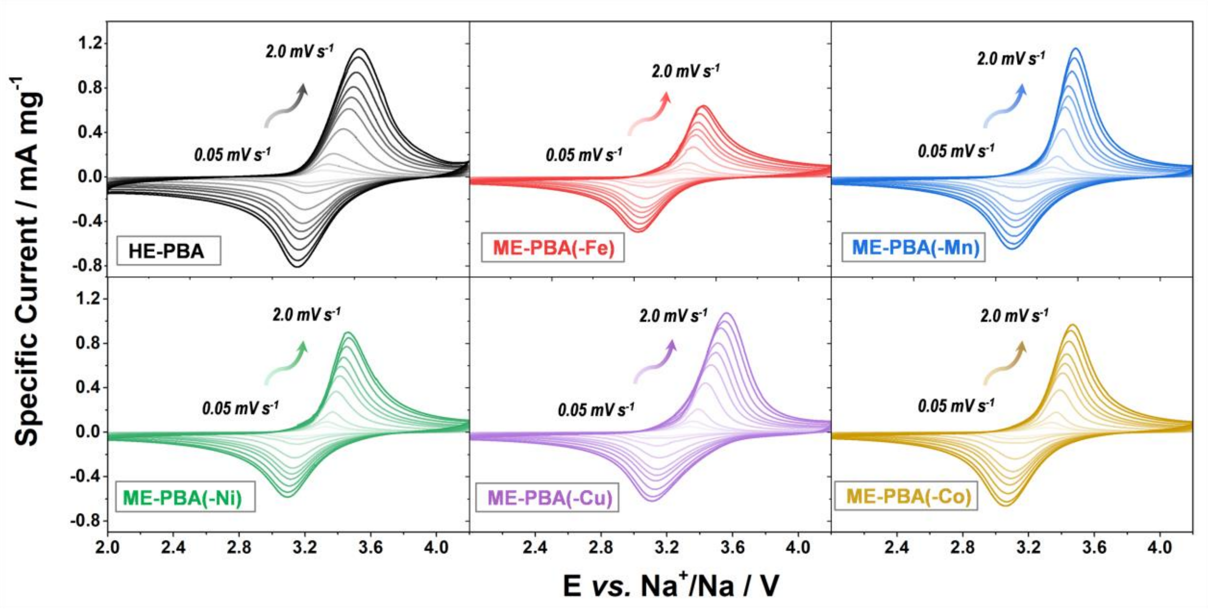

Figure S11. Cyclic voltammograms at different sweep rates ranging from 0.05 to 2.0 mV s^{-1} for electrodes based on HE-PBA (in black), ME-PBA(-Fe) (in red), ME-PBA(-Mn) (in blue), ME-PBA(-Ni) (in green), ME-PBA(-Cu) (in purple) or ME-PBA(-Co) (in dark yellow).

Calculation of apparent diffusion coefficient

The sodium-ion diffusion coefficient was calculated by analyzing the peak current (I_p) from cyclic voltammetry measurements (**Figure S11**) using the Randles-Sevcik equation:[26]

$$I_{\mathrm{p}} = 2.68 \cdot 10^5 n^{3/2} A D^{1/2} C \omega^{1/2}, \qquad \textbf{(Equation S3)}$$

where n is the number of involved electrons, A is the contact area between the electrode material and electrolyte (here the geometric area of the electrode is used for simplicity, cm^2), D is the apparent diffusion coefficient ($cm^2\ s^{-1}$), C is the molar concentration (mol cm^{-3}) of sodium ions in PBA and ω (mV s^{-1}) represents the sweep rate. The corresponding values are given in **Table S5**.

Table S5. Apparent sodium-ion diffusion coefficient in units of $cm^2\ s^{-1}$ for HE-PBA-, ME-PBA(-Fe)-, ME-PBA(-Mn)-, ME-PBA(-Ni)-, ME-PBA(-Cu)- and ME-PBA(-Co)-based electrodes.

	**HE-PBA**	**ME-PBA(-Fe)**	**ME-PBA(-Mn)**	**ME-PBA(-Ni)**	**ME-PBA(-Cu)**	**ME-PBA(-Co)**
**De-sodiation**	$2.07 \cdot 10^{-8}$	$6.61 \cdot 10^{-9}$	$1.95 \cdot 10^{-8}$	$1.23 \cdot 10^{-8}$	$1.78 \cdot 10^{-8}$	$1.45 \cdot 10^{-8}$
**Re-sodiation**	$9.75 \cdot 10^{-9}$	$3.70 \cdot 10^{-9}$	$6.37 \cdot 10^{-9}$	$4.99 \cdot 10^{-9}$	$5.87 \cdot 10^{-9}$	$6.84 \cdot 10^{-9}$

Table S6. Comparison of the structural changes of HE-PBA and PBAs reported in the literature upon de-/sodiation.

**Material**	**De-sodiation ⇌ Re-sodiation**	**Reference**
**HE-PBA**	**Cubic**	**This work**
MnFe-PBA	Monoclinic ⇌ Cubic ⇌ Tetragonal	[27][28][29]
MnFe-PBA	Rhombohedral ⇌ Rhombohedral + Na-poor phase ⇌ Tetragonal	[28]
NiFe-PBA	Rhombohedral ⇌ Cubic	[30]
CuFe-PBA	Cubic ⇌ Rhombohedral	[11]
MnFe-PBA	Monoclinic ⇌ Cubic	[18]
MnCoFe-PBA	Rhombohedral ⇌ Cubic	[31]
NiCoFe-PBA	Monoclinic ⇌ Cubic ⇌ Tetragonal	[23]

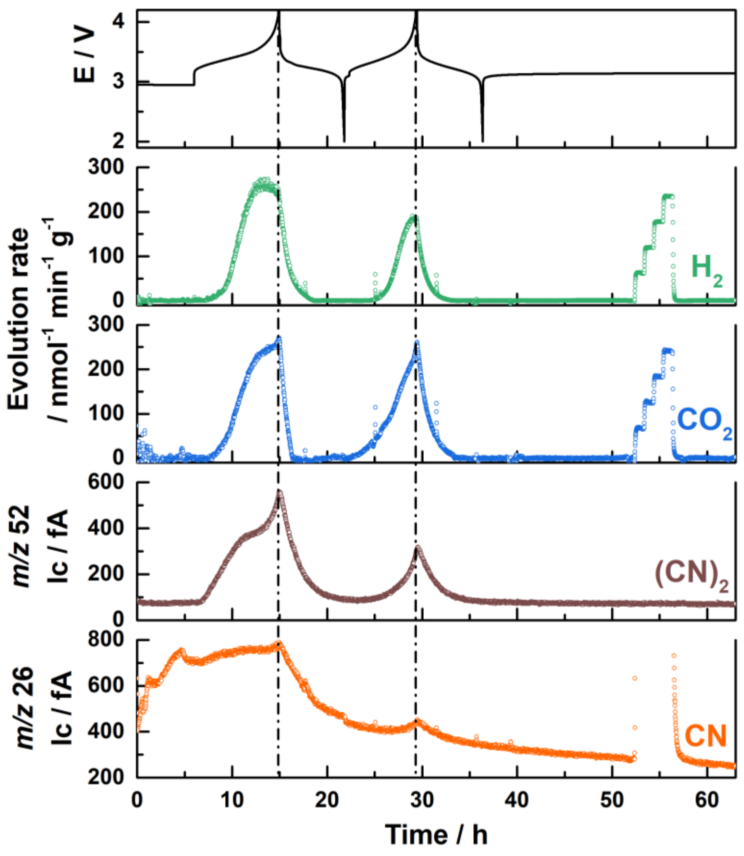

Figure S12. Gassing behavior of the HE-PBA cathode during de-/sodiation in the first two cycles probed using DEMS. Evolution rates of H_2 (green) and CO_2 (blue) are shown. For *m*/*z* = 52 (brown) and 26 (orange), only ion currents are given (no calibration standard available). Note that the *m*/*z* = 26 signal during calibration stems from ethylene in the calibration gas mixture.

References

[1] B. Ravel, M. Newville, *Journal of Synchrotron Radiation* **2005**, *12*, 537.

[2] B. B. Berkes, A. Jozwiuk, H. Sommer, T. Brezesinski, J. Janek, *Electrochemistry Communications* **2015**, *60*, 64.

[3] B. B. Berkes, A. Jozwiuk, M. Vračar, H. Sommer, T. Brezesinski, J. Janek, *Analytical Chemistry* **2015**, *87*, 5878.

[4] B. S. Murty, J.-W. Yeh, R. Srikanth, P. P. Bhattacharjee, *High-Entropy Alloys*, Elsevier, **2019**.

[5] M. C. Gao, J. Yeh, P. K. Liaw, Y. Zhang, *High-Entropy Alloys*, Cham: Springer International Publishing, **2016**.

[6] J. C. Pramudita, S. Schmid, T. Godfrey, T. Whittle, M. Alam, T. Hanley, H. E. A. Brand, N. Sharma, *Physical Chemistry Chemical Physics* **2014**, *16*, 24178.

[7] D. B. Brown, D. F. Shriver, L. H. Schwartz, *Inorganic Chemistry* **1968**, *7*, 77.

[8] H. Fu, C. Liu, C. Zhang, W. Ma, K. Wang, Z. Li, X. Lu, G. Cao, *Journal of Materials Chemistry A* **2017**, *5*, 9604.

[9] S. Yu, Y. Li, Y. Lu, B. Xu, Q. Wang, M. Yan, Y. Jiang, *Journal of Power Sources* **2015**, *275*, 45.

[10] D. Yang, J. Xu, X. Z. Liao, Y. S. He, H. Liu, Z. F. Ma, *Chemical Communications* **2014**, *50*, 13377.

[11] Z. Wang, Y. Huang, R. Luo, F. Wu, L. Li, M. Xie, J. Huang, R. Chen, *Journal of Power Sources* **2019**, *436*, 226868.

[12] M. Xie, M. Xu, Y. Huang, R. Chen, X. Zhang, L. Li, F. Wu, *Electrochemistry Communications* **2015**, *59*, 91.

[13] W. Li, C. Han, W. Wang, Q. Xia, S. Chou, Q. Gu, B. Johannessen, H. Liu, S. Dou, *Advanced Energy Materials* **2020**, *10*, 1903006.

[14] Y. Liu, D. He, R. Han, G. Wei, Y. Qiao, *Chemical Communications* **2017**, *53*, 5569.

[15] Y. Huang, M. Xie, J. Zhang, Z. Wang, Y. Jiang, G. Xiao, S. Li, L. Li, F. Wu, R. Chen, *Nano Energy* **2017**, *39*, 273.

[16] L. Wang, Y. Lu, J. Liu, M. Xu, J. Cheng, D. Zhang, J. B. Goodenough, *Angewandte Chemie International Edition* **2013**, *52*, 1964.

[17] W. J. Li, S. L. Chou, J. Z. Wang, Y. M. Kang, J. L. Wang, Y. Liu, Q. F. Gu, H. K. Liu, S. X. Dou, *Chemistry of Materials* **2015**, *27*, 1997.

[18] X. Bie, K. Kubota, T. Hosaka, K. Chihara, S. Komaba, *Journal of Power Sources* **2018**, *378*, 322.

[19] S. Jiao, J. Tuo, H. Xie, Z. Cai, S. Wang, J. Zhu, *Materials Research Bulletin* **2017**, *86*, 194.

[20] M. J. Piernas-Muñoz, E. Castillo-Martínez, O. Bondarchuk, M. Armand, T. Rojo, *Journal of Power Sources* **2016**, *324*, 766.

[21] Y. Lu, L. Wang, J. Cheng, J. B. Goodenough, *Chemical Communications* **2012**, *48*, 6544.

[22] Y. Tang, W. Li, P. Feng, M. Zhou, K. Wang, Y. Wang, K. Zaghib, K. Jiang, *Advanced Functional Materials* **2020**, *30*, 1908754.

[23] J. Peng, J. Wang, H. Yi, W. Hu, Y. Yu, J. Yin, Y. Shen, Y. Liu, J. Luo, Y. Xu, P. Wei, Y. Li, Y. Jin, Y. Ding, L. Miao, J. Jiang, J. Han, Y. Huang, *Advanced Energy Materials* **2018**, *8*, 1702856.

[24] P. Ge, S. Li, H. Shuai, W. Xu, Y. Tian, L. Yang, G. Zou, H. Hou, X. Ji, *Advanced Materials* **2019**, *31*, 1806092.

[25] L. Li, P. Nie, Y. Chen, J. Wang, *Journal of Materials Chemistry A* **2019**, *7*, 12134.

[26] X. Hou, Y. Huang, S. Ma, X. Zou, S. Hu, Y. Wu, *Materials Research Bulletin* **2015**, *63*, 256.

[27] Y. Shang, X. Li, J. Song, S. Huang, Z. Yang, Z. J. Xu, H. Y. Yang, *Chem* **2020**, *6*, 1804.

[28] J. Song, L. Wang, Y. Lu, J. Liu, B. Guo, P. Xiao, J. J. Lee, X. Q. Yang, G. Henkelman, J. B. Goodenough, *Journal of the American Chemical Society* **2015**, *137*, 2658.

[29] J. Sottmann, F. L. M. Bernal, K. V. Yusenko, M. Herrmann, H. Emerich, D. S. Wragg, S. Margadonna, *Electrochimica Acta* **2016**, *200*, 305.

[30] W. Ren, M. Qin, Z. Zhu, M. Yan, Q. Li, L. Zhang, D. Liu, L. Mai, *Nano Letters* **2017**, *17*, 4713.

[31] M. Pasta, R. Y. Wang, R. Ruffo, R. Qiao, H. W. Lee, B. Shyam, M. Guo, Y. Wang, L. A. Wray, W. Yang, M. F. Toney, Y. Cui, *Journal of Materials Chemistry A* **2016**, *4*, 4211.
